# Teaching tools in Evidence Based Practice: evaluation of reusable learning objects (RLOs) for learning about Meta-analysis

**DOI:** 10.1186/1472-6920-11-18

**Published:** 2011-05-04

**Authors:** Fiona Bath-Hextall, Heather Wharrad, Jo Leonardi-Bee

**Affiliations:** 1 School of Nursing, Midwifery & Physiotherapy, University of Nottingham, Nottingham, UK; 2 Division of Epidemiology and Public Health, University of Nottingham, Nottingham, UK

## Abstract

**Background:**

All healthcare students are taught the principles of evidence based practice on their courses. The ability to understand the procedures used in systematically reviewing evidence reported in studies, such as meta-analysis, are an important element of evidence based practice. Meta-analysis is a difficult statistical concept for healthcare students to understand yet it is an important technique used in systematic reviews to pool data from studies to look at combined effectiveness of treatments. In other areas of the healthcare curricula, by supplementing lectures, workbooks and workshops with pedagogically designed, multimedia learning objects (known as reusable learning objects or RLOs) we have shown an improvement in students' perceived understanding in subjects they found difficult. In this study we describe the development and evaluation of two RLOs on meta-analysis. The RLOs supplement associated lectures and aim to improve students' understanding of meta-analysis in healthcare students.

**Methods:**

Following a quality controlled design process two RLOs were developed and delivered to two cohorts of students, a Master in Public Health course and Postgraduate diploma in nursing course. Students' understanding of five key concepts of Meta-analysis were measured before and after a lecture and again after RLO use. RLOs were also evaluated for their educational value, learning support, media attributes and usability using closed and open questions.

**Results:**

Students rated their understanding of meta-analysis as improved after a lecture and further improved after completing the RLOs (Wilcoxon paired test, p < 0.01 in all cases) Whilst the media components of the RLOs such as animations helped most students (86%) understand concepts including for example Forest plots, 93% of students rated usability and control as important to their learning. A small number of students stated they needed the support of a lecturer alongside the RLOs (7% 'Agreed' and 21% 'Neutral').

**Conclusions:**

Meta-analysis RLOs that are openly accessible and unrestricted by usernames and passwords provide flexible support for students who find the process of meta-analysis difficult.

## Background

*"All health care professions need to have an understanding of EBP, understand the principles of evidence based practice (EBP), recognise EBP in action, implement evidence-based policies, and have a critical attitude to their own practice and to evidence. Without these skills, professionals and organisations will find it difficult to provide best practice" *[1]

Evidence based medicine is described as "the conscientious, explicit and judicious use of current best evidence in making decisions about the care of individual patients" [2]. However research based information is not used in isolation but together with patient preference and an individual's knowledge and expertise. Similarly, evidence based nursing can be defined as the application of valid, relevant, research-based information in nurse decision-making [3]. Standards of conduct, performance and ethics for nurses and midwives state that nurses and midwives 'must deliver care based on the best available evidence or best practice' [4]. To be able to do this a knowledge of evidence based practice (EBP) is required and the skills to perform EBP.

Whilst these definitions recognise the importance of individual clinical expertise and patient choice as determinants of clinical decision making, the randomised trial is regarded as the gold standard for judging whether a treatment is beneficial. The volume of data that needs to be considered by practitioners is constantly expanding and keeping up to date with literature evidence can be a challenge. Therefore, reviews have become essential tools to keep up to date with new evidence since they collate and evaluate primary research on a focused topic. *Systematic *reviews allow for a more objective appraisal of the evidence compared to traditional literature reviews and a technique called *meta-analysis *is commonly used in scientific papers and systematic reviews looking at effectiveness of treatments. Meta-analysis is a statistical technique for pooling the results from similar studies in order to increase the statistical power and therefore is an important concept to get to grips with in EBP.

In the School of Nursing, Midwifery & Physiotherapy at the University of Nottingham, EBP is an integral part of the curriculum for pre registration through to post registration and postgraduate education courses. EBP is also a component of the undergraduate Medical courses and the Master in Public Health programme. Lecturers on these courses recognised from assignments and assessments that students did not fully understand the statistical technique of meta-analysis or the various important concepts that underpin meta-analysis.

Whilst lectures result in delivery of information they do not necessarily engender learning and understanding which may be better supported by blended or more applied teaching methodologies. Our previous studies of students in health sciences (particularly in areas of the curriculum that they find difficult) have shown that by supplementing lectures with e-learning resources that are interactive, visual, and small in size and highly aligned with their perceived learning needs [5,6] improve their understanding and attainment [7,8].

In view of this, we wanted to enhance students learning of meta-analysis by designing and developing flexible e-learning tools in the form of two reusable learning objects (RLOs) to accompany the usual didactic lectures. An evaluation study with healthcare students would determine the impact of the RLOs on their perceived understanding of meta-analysis concepts and views on usability and design of the e-learning materials.

Numerous definitions for an RLO exist [9,10] however our definition of an RLO is: 'an interactive, multimedia web-based resource based on a single learning objective which can be used in multiple contexts'. Basically, they are bite sized chunks of e-learning, focusing on a specific topic. They are highly visual with an auditory component and high quality graphics and take the average student about 15 minutes to complete.

The aims of this study were:

1. To design and develop two RLOs on meta-analysis

2. To evaluate the educational and media attributes of the RLOs with students on a postgraduate diploma in nursing (PGN) course and a Master's in Public Health (MPH) course in a blended learning setting.

3. To compare the self-reported ratings of understanding of five key elements of meta-analysis in the two student groups.

We will also report briefly on re-use of the RLOs by other students outside of the study group for whom the RLOs were originally designed for.

## Methods

### RLO development and quality control process

**Figure 1:**
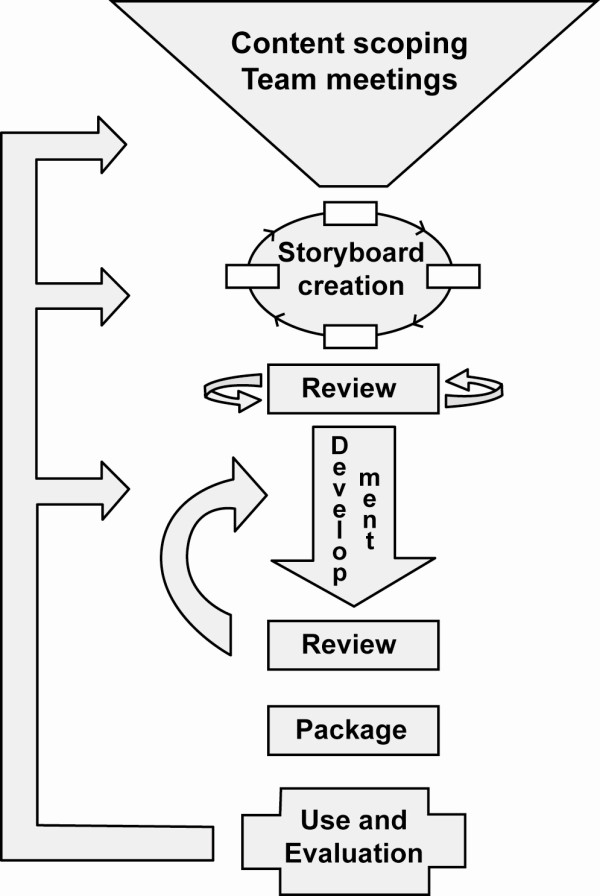
**Schematic diagram showing the stages of the RLO development process**. The schematic diagram shows the stages of the RLO development process beginning with team meetings to scope the content and ideas for analogies and media to illustrate the concepts. The written storyboard is sent to experts for peer review prior to development. Once a prototype has been developed, the RLOs go through a second peer review before packaging and release.

Figure 1 outlines the process for developing RLOs; this was based on a well established methodology [11].

**Figure 2:**
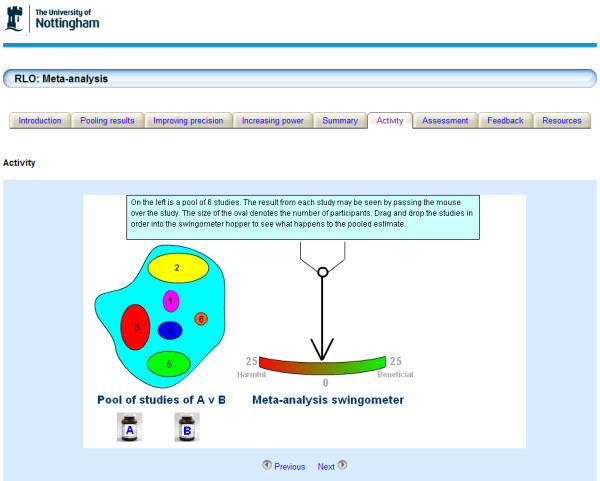
**Screen shot from the Introduction to Meta-analysis RLO**. Screen shot from the Introduction to Meta-analysis RLO. The RLO is divided into sections each having a tabbed heading. RLOs contain animations, activities and self assessments.

**Figure 3:**
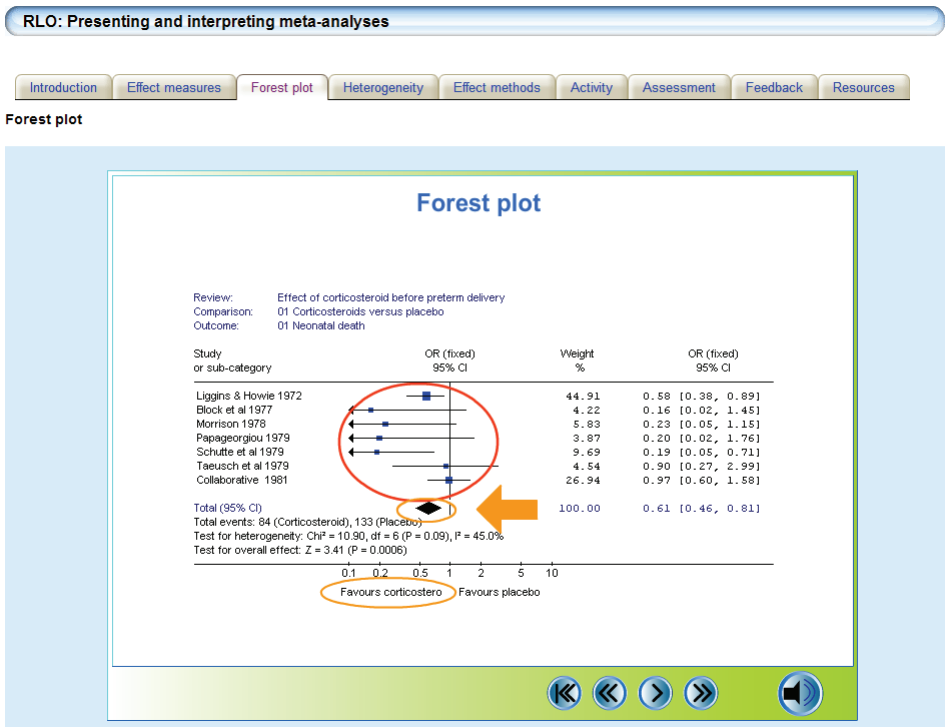
**Screen shot from the Presenting and interpreting Meta-analysis RLO**. Screen shot from the Presenting and interpreting Meta-analysis RLO. This RLO covers effect measures, forest plots, heterogeneity and effect methods.

Storyboards (written templates of the proposed content) were developed through an iterative development cycle. Academics in the University, from different disciplines, assessed the accuracy and ease of understanding of the RLOs through an iterative internal peer review process (two experts review the storyboard for accuracy and appropriateness of the content recording their responses onto a structured proforma). After peer review the RLOs were assembled and then released for a second internal peer review by experts and evaluation by students (in this second peer review, the reviewers are assessing usability and appropriateness of media to explain the concepts). The first RLO provided an introduction to meta-analysis, sections covered pooling results, improving precision and improving power; the second RLO was more detailed covering effect measures, forest plots, heterogeneity and effect methods. Both RLOs incorporated activities and a self assessment activity to provide formative feedback on learning (Figures 2 and 3).

The RLOs are freely and openly accessible under a Creative Commons licence on the University of Nottingham SONET website http://www.nottingham.ac.uk/nursing/sonet/rlos/ebp/meta-analysis/

http://www.nottingham.ac.uk/nursing/sonet/rlos/ebp/meta-analysis2/

### Study sample and setting

The PGN course was a diploma level nursing course for graduates (whose first degrees were across the whole range of disciplines). On successful completion of the PGN, students qualified with a nursing registration. The MPH course was a master's level course where students came from a range of interprofessional health care backgrounds including medicine, nursing and physiotherapy. In each case, students received their usual lecture on meta-analysis and were then given time to work through the two RLOs in a computer lab. All students attending the meta-analysis sessions took part in the study therefore the student number was a convenience sample.

### Ethical Considerations

The study comes within the category 'educational evaluation' within our institution and therefore does not require full ethical approval. These studies do require students to be provided with information about what they are being asked to do and to provide consent (with the option of withdrawing from the study). Students were aware that they were taking part in an evaluation study and that the data they provided was anonymous and that the study might be published.

### Evaluation process and tools

(i) A questionnaire adapted from a previous study [7], was used to assess the students' perceived understanding of five different elements of meta-analysis. They were asked to rate their understanding of (i) what meta- analysis was; (ii) effect measures; (iii) forest plots; (iv) heterogeneity and (v) effects methods. Ratings were made on an ordinal scale ranging from 1 to 10 where 1 is I understand 'very well' and 10 is 'very badly'. A 10 point numerical scale was used rather than a 4 or 5 point numerical or text scale to reduce the possibility that students would remember their prior responses thus biasing the results. Students could not refer to their previous ratings and the questionnaires were analysed by an independent researcher not the students' lecturers. The questionnaire was administered prior to the standard lecture on meta-analysis, again after the lecture and then after accessing the RLOs. Access to the RLOs was only given after the lead lecture. The time between administering the first and last questionnaire was several hours. Students were encouraged to complete the three open response text boxes at the end of the RLO asking 'Will you access these RLOs again?', 'Where will you access the RLOs from?', 'How might you use the knowledge about meta-analysis in your future practice?'. Analysis of these qualitative data is described below.

(ii) Students' evaluations of the RLOs were measured using a toolkit devised by the Centre for Excellence in Teaching and Learning for Reusable Learning Objects http://www.rlo-cetl.ac.uk. This evaluation strategy is based on Activity Theory and has been widely deployed [12]. Two tools were employed. Firstly, a short online user feedback form presented at the end of each RLO which asked the students to rate the RLO as learning tools (for example 'How easy was it to use the learning object?') using 4-point Likert scales and two open questions asking students to comment on what they liked and suggestions for improving the RLO. Secondly, a paper questionnaire containing Likert ratings and open questions relating to the use and delivery of the RLOs as a collection was handed to each student. Evidence of reuse of the objects by other learners was gained from analysis of online feedback forms (described above) submitted by individuals outside of the target cohorts at the focus of this study.

### Data Analysis

Quantitative data: Online feedback data was collated automatically within the survey management tool Zoomerang http://www.zoomerang.com and was exported into Microsoft Excel. Data from the paper questionnaire were entered into a Statistical Package for the Social Sciences (SPSS vs. 15); descriptive statistics and frequency tables were calculated. Within group responses for the PGN and the MPH students were compared using the non parametric signed rank Wilcoxon paired test. Between group responses were compared using Mann Whitney test. The level of significance was set at p < 0.05.

Qualitative data: Open ended responses were organised into key themes by one of the authors, these were shared among the other authors for verification. Quotations representing the key themes were later selected and used alongside literature evidence to illustrate the key issues.

## Results

### Change in student understanding

**Figure 4:**
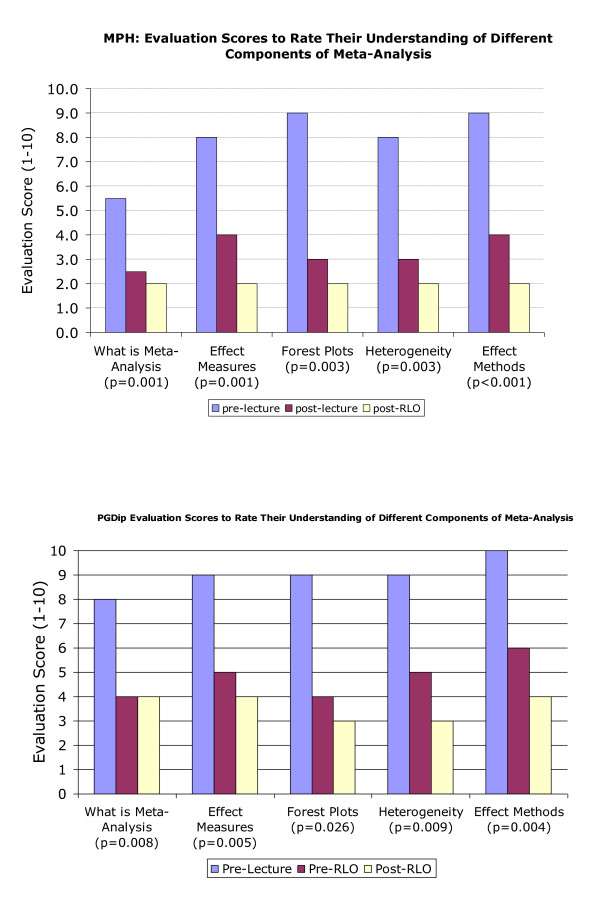
**Masters in Public Health (MPH) and Post graduate diploma (PGD) students self reported rating of their understanding of different aspects of Meta-analysis**. Masters in Public Health (MPH) and Post graduate diploma (PGD) students self reported rating of their understanding of different aspects of Meta-analysis rated on an ordinal scale of 1 to 10 where 1 is 'Very Well' and 10 is 'Very Badly'. The p values are for the difference in perceived understanding following RLO use (post RLO - cream bars) compared to the rating before the RLO and after the lecture (pre-RLO purple bars).

Figure 4 shows the mean ratings of 'understanding' (on a scale of 1 - 'very well' to 10 'very badly') for five important components of meta-analysis. Perceived understanding improved both after the lecture and again after the RLOs. For both MPH (n = 26) and PGD (n = 12) students' self-reported understanding of all five aspects of meta-analysis improved significantly (Wilcoxon paired p < 0.05 in all cases). The p values are for the difference in perceived understanding following RLO use (post RLO) compared to the rating before the RLO and after the lecture (pre-RLO).

There were no significant differences between the two student groups understanding (Mann Whitney p > 0.05). There were no missing values in the data set.

### RLO evaluation and usability

Table 1 shows the student ratings of a range of attributes of the RLOs (responses for the two groups have been combined since there were no significant differences between them). In terms of educational value, responses overall were positive, 93% of students agreed or strongly agreed with the statement 'The RLO has aided my understanding and I feel I have achieved the learning objective' with slightly fewer (71%) agreeing or strongly agreeing with the statement 'I am confident that I will be able to use the knowledge gained from this RLO in future practice'

**Table 1:** Student ratings of attributes of Meta-analysis RLOs divided into five categories, Educational value, Learning support, Flexibility and support, Usability and Media attributes.

Educational Value	Strongly Agree	Agree	Neutral	Disagree	Strongly Disagree	Missing Data
The content was appropriate and fitted my learning needs	21%	64%	7%	0%	7%	0

The activity was appropriate and aided my understanding	36%	43%	14%	0%	7%	0

The RLO encouraged me to reflect on the material	29%	36%	29%	0%	7%	0

I am confident that I will be able to use the knowledge gained from this RLO in future practice	21%	50%	21%	0%	0%	1

The RLO has aided my understanding and I feel I have achieved the learning objective.	36%	57%	0%	0%	7%	0

The RLO will help me retain information	29%	43%	14%	0%	0%	2

The self-assessment helped me gauge how well I'd understood the material	43%	14%	21%	0%	0%	3

I will use this RLO again	36%	21%	36%	0%	7%	0

The RLO integrated well with the module and other teaching sessions	50%	43%	0%	0%	0%	1

**Learning support**						

The RLO was interesting and engaging	36%	50%	14%	0%	0%	0

I needed the help of a lecturer to understand the content	0%	7%	21%	50%	21%	0

The RLO was pitched at the right level for me	21%	57%	7%	7%	7%	0

I needed more support when using the RLO	7%	7%	7%	36%	43%	0

**Flexibility and control**						

I enjoyed being able to work at my own pace	57%	14%	14%	0%	0%	2

I like the idea that I can access this RLO whenever I want to	79%	14%	0%	0%	7%	0

**Usability**						

The RLO was well structured and easy to follow	50%	43%	7%	0%	0%	0

The RLO was easy to use	64%	29%	7%	0%	0%	0

The RLO was easy to navigate I felt in control.	50%	50%	0%	0%	0%	0

I liked the look and feel of the RLO	21%	71%	7%	0%	0%	0

**Media attributes **						

The images and animations were valuable components of the RLO	57%	29%	0%	14%	0%	0

The on-screen text was useful and helped me assess the amount of information each section contained.	36%	36%	29%	0%	0%	0

The RLO took longer to complete than expected	0%	7%	36%	57%	0%	0

The narration made the RLO more engaging. I preferred this to text alone	43%	29%	7%	7%	7%	1

Responses from PGN and MPH groups have been combined (n = 38).

Under the category Learning Support, students mostly disagreed that they needed more support when using the RLOs (79%). Ratings for usability were higher than for any other category however under Media attributes, there were a few respondents (14%) who were less positive about the value of the images and animations, and the narration.

### Open questions

Table 2 outlines the key themes emerging from the responses to the open questions. Five broad themes (T1-5) were identified from the students comments - (T1) need for lecturer support, (T2) the value of the interactivity and animations, (T3) the level of detail in the RLOs and the timing of when they are delivered in relation to lectures (T4) discoverability of the RLOs and (T5) catering for different learning styles.

**Table 2:** Themes identified from the open ended questions on the online feedback from and the paper based questionnaire.

Theme	Student Quotations
T1. Lecturer support	*"Easy diagrammatic format. I am not so sure how meaningful and easy it would be to follow without prior explanation [of the concepts] from tutor"*

T2. Value of interactivity and animations	*"The use of a pool and balance to explain the process rather than a forest plot"* *"Generally I liked all that was presented. Was impressed by the meter as it helps to explain the whole concept"* *"Very clear and concise. It helped me understand much better than my text. The learning activities reinforced my understanding. Thank you"* *"I liked the interactive summaries which simplified and demonstrated what the reader was talking about- very visual and easy to understand"* *"Its interactive nature and use of simple words"*

T3. Level and timing in relation to lectures	*"I would have wanted to have more exercises. Although this would make the RLO longer to get through, it would be more worthwhile."* *"Over-laps lecture - maybe be beneficial to use before the lecture as a basic introduction"* *"A bit basic following the lectures. Be better to be introduced to it before the teaching session"* *"Your lecture was better, and was in much greater depth. I liked the speaking rather than reading, but did not find that the pictures added much, particularly as they often repeated the same percentage changes in spiralling boxes"* *"The support for the lecture we received was good, and points that confused me in the lecture I could take my time over and complete"*

T4. Resource discovery	*"RLO was very useful, its more a case of being aware (or reminded) that they exist"* *"Very clear, easy to understand. Should have checked it out earlier!"*

T5. Learning style	*"combining sound and visual is good for visual learners. The quiz helped consolidate"* *"Visual learning is always best for me."* *"The narration it reinforced concepts introduced in lecture"*

Quotations have been provided to represent the themes.

For the two RLOs, to date there have been 485 visits to the online feedback forms, this is the number of users who have completed the RLO of these 43 (16%) and 49 (22%) completed the feedback form for Introduction to Meta-analysis and Presenting and interpreting meta-analysis respectively. 62 respondents were Nottingham based and 27 non-Nottingham (and over 50% of these non-UK, from US, Australia, India, New Zealand, Thailand and Brazil). All respondents rated the RLOs as excellent or good and there were no significant differences between ratings of University of Nottingham and non-Nottingham respondents (Wilcoxon paired p > 0.05).

## Discussion

The development of RLOs on meta-analysis was in response to lecturers on healthcare courses recognising from assignments and assessments that students did not fully understand the statistical technique of meta-analysis or the various important concepts that underpin meta-analysis. Students reported they felt their understanding had been improved by the RLOs (Figure 4) and there were certain features of the RLOs described in Tables 1 and 2 that appeared to contribute to this enhancement by adding value to lecture delivery. These findings are supported by other studies describing the educational benefits of RLOs in blended learning settings in healthcare curricula [7,8] and improved learning performance in clinical laboratory sciences [13]. Generally, RLOs have been identified as having an important role to play in new medical curricula abroad [14,15].

Visual learning approaches have been shown to enhance learning by providing multiple representations of a topic and by supporting learner preferences [16,17]. The visual and interactive elements along with the use of analogies to describe the meta-analysis process seemed to contribute to the students' perceived improvement in understanding as illustrated by the quotes (T2) in Table 2. A recent report also alludes to the importance of appropriate visual representation describing an approach using simplified graphics of Forest plots to increase nurses' awareness of effect measures for different levels of data (adjusted means for continuous data and odds ratios for discrete data). These authors however provide only anecdotal evidence of the usefulness of these simplified plots [18].

A criticism directed at RLOs is their instructivist approach [19]. We would argue that pedagogical strategies to encourage active learning can be built into the RLO design and how the RLOs are integrated into a course determines their value in promoting reflection and deeper learning. Both the value of self assessment and reflection on the material were highly scored by the students (Table 1 Educational Value). Further research is needed to test the assertion that the use of RLOs can lead to deeper learning and the context of use of the RLOs will be an important variable to consider.

Boyle and Cook [20] suggest that RLOs should fit a cohesion and decoupling model referring to the idea that they are self contained learning units and the content is aligned with the learning goal and assessment and free from external links. The feedback suggests that some students would have struggled with the content of the RLO using it alone as a replacement for the didactic lecture and they would need the support of a teacher (Table 1 Learning Support; Table 2 T1, T3). Some students however wanted more detail and felt that the RLO should be offered prior to the lecture. Whilst the pedagogical design of the RLOs provides the flexibility for them to be used as stand alone units, most students still prefer RLOs to support blended delivery [7,8].

The similar positive responses of the PGD and MPH students suggest that the flexibility of the utility of RLOs makes them suitable for multiprofessional learning perhaps providing support for having shared open repositories for healthcare education [15]. The framework for the design process [11] certainly caters for multiprofessional debate amongst healthcare educators around content and associated learning activities [21].

Previous investigations of the effectiveness of e-learning technologies for health professionals identified a number of barriers to its success among them cost, poorly designed packages, lack of skills, need for a component of face-to-face teaching, time intensive nature of e-learning and computer anxiety [6,22]. This study has shown how many of these factors have been addressed. One of the reasons for making our resources freely and openly accessible were the difficulties students had accessing materials requiring usernames and passwords and remembering to access them. Two students alluded to this in their comments (Table 2 T4).

With the plethora of open educational resources now available to students, there are a new set of issues around 'discovery' and 'filtering' to find the good quality pedagogically defined learning materials.

The value of these meta-analysis RLOs may be partly because they are by their very nature different to traditional e-learning tools. Our data suggest that individual RLOs do not require more than around 15 minutes to complete thus they do not require a time-intensive input making them more flexible for students to use at work or home, attributes previously shown to be valued by students [5,22]. Ratings of flexibility and control were rated more highly than the value of the media attributes (Table 1 Flexibility and Control vs Media Attributes), this has been reported before in e-learning studies [7,8,23]. It is asserted that a sense of ownership and control over learning is important for healthcare students coping with busy curricula and work placements [6,8]. Learners taking more active control of visual learning approaches construct a deeper understanding of the subject [24].

The visual, audio and interactive nature of these RLOs means that they have an appeal for visual, auditory and kinesthetic learners an important issue bearing in mind data which suggests that learning style is important in web-based e-learning [9,25].

However, not all students liked the RLO or some of the media used, though these students were in the minority (Table 1).

In terms of the usability and media attributes of the RLOs the majority of students

either agreed or strongly agreed with statements relating to the ease of use of the RLOs and the value of the different components which make up the RLO reinforcing their importance in enhancing learning and understanding (Table 1 Usability and Media Attributes).

The 'not invented here' view has been suggested to be a barrier to the reuse of resources [26] because lecturers want to make them 'their own' and contextualise the resource for their particular students. This does not seem to be a problem for students however since we found no differences in the RLO ratings for Nottingham students compared to non-Nottingham students. A non-Nottingham student valued "The step-by-step description of each part of the Forest plot as well as basic information imparted by each tab given".

### Limitations

This was a small scale evaluation study based on a convenience sample that relied on students' self-reporting their levels of understanding. Objective measures of improvement in knowledge of meta-analysis were not collected because students agreed to take part in the study on the basis that their responses were anonymous and would not be followed up in summative assessments.

## Conclusions

Understanding meta-analysis will give confidence in reading scientific papers and systematic reviews which in turn will encourage evidence-based practice ultimately leading to improved patient care. The RLOs were successful in supporting the students understanding of meta-analysis, resulting in a perceived increase in understanding of the various components of meta-analysis. The RLOs evaluated extremely positively by the students, and the students reported that they continued to access the RLOs after the evaluation. RLOs are used flexibly; not only to help students with difficult topics but also to illustrate concepts or skills. Students use them to plug gaps in their knowledge and to prepare for lectures/tutorials or as revision aids. The feedback showed that the RLOs supported learning but students still valued the lectures therefore we recommend that RLOs are used as a supplement but not to replace other delivery approaches. This is the first study to report on the evaluation of meta-analysis resources.

## Competing interests

The authors declare that they have no competing interests.

## Authors' contributions

FB-H and JL-B were involved in the conception and design, writing of the storyboards, acquisition of data, analysis and interpretation of data; and in drafting the manuscript and revising it critically for important intellectual content. HW was involved in the analysis and interpretation of data; and in re-drafting the manuscript and revising it critically for important intellectual content. All authors read and approved the final manuscript. This work formed part of J L-B's Post Graduate Certificate in Higher Education qualification.

## Pre-publication history

The pre-publication history for this paper can be accessed here:

http://www.biomedcentral.com/1472-6920/11/18/prepub
